# Recent Advancements in the Regeneration of Auditory Hair Cells and Hearing Restoration

**DOI:** 10.3389/fnmol.2017.00236

**Published:** 2017-07-31

**Authors:** Rahul Mittal, Desiree Nguyen, Amit P. Patel, Luca H. Debs, Jeenu Mittal, Denise Yan, Adrien A. Eshraghi, Thomas R. Van De Water, Xue Z. Liu

**Affiliations:** ^1^Department of Otolaryngology, University of Miami Miller School of Medicine Miami, FL, United States; ^2^Department of Otolaryngology, Xiangya Hospital, Central South University Changsha, China

**Keywords:** auditory hair cells, hair cell regeneration, hearing loss, gene therapy, stem cell therapy

## Abstract

Neurosensory responses of hearing and balance are mediated by receptors in specialized neuroepithelial sensory cells. Any disruption of the biochemical and molecular pathways that facilitate these responses can result in severe deficits, including hearing loss and vestibular dysfunction. Hearing is affected by both environmental and genetic factors, with impairment of auditory function being the most common neurosensory disorder affecting 1 in 500 newborns, as well as having an impact on the majority of elderly population. Damage to auditory sensory cells is not reversible, and if sufficient damage and cell death have taken place, the resultant deficit may lead to permanent deafness. Cochlear implants are considered to be one of the most successful and consistent treatments for deaf patients, but only offer limited recovery at the expense of loss of residual hearing. Recently there has been an increased interest in the auditory research community to explore the regeneration of mammalian auditory hair cells and restoration of their function. In this review article, we examine a variety of recent therapies, including genetic, stem cell and molecular therapies as well as discussing progress being made in genome editing strategies as applied to the restoration of hearing function.

## Introduction

Hearing loss is a serious global morbidity that affects 360 million people worldwide (WHO, 2013). Hearing loss is a neurological disability that impacts both the physical and mental well-being of patients (Contrera et al., 2016; Roland et al., 2016; Goman et al., 2017; Homans et al., 2017). At the basic level, loss of hearing is a source of isolation and depression for patients due to the importance of hearing in communication (Li et al., 2014; Sun et al., 2016; Tseng et al., 2016). Individuals with hearing disabilities often have to rely more heavily on caretakers. Patients with hearing loss are at increased risk for accidental injury due to their loss of this sense (Smith, 2015). Children with hearing loss are at danger of diminished neurological cognitive development as a result of their reduced exposure to sound stimuli and language (Boulet et al., 2009; Stevenson et al., 2015; Niclasen et al., 2016; Roland et al., 2016). Hearing loss represents a profound burden for both patients and the medical system (WHO, 2013; Olusanya et al., 2014).

Hearing loss displays a distribution that correlates with geographic location and socioeconomic trends (Helvik et al., 2009; Emmett and Francis, 2015; Qing et al., 2015; Yan et al., 2015, 2016, 2017). Geographically, hearing loss is most common in Sub-Saharan Africa, the Asia Pacific, and Southern Asia (Yan et al., 2015). Socioeconomic status is inversely correlated with hearing loss. The children from low gross national income (GNI) nations display a 2% greater prevalence of hearing loss compared to the incidence of hearing loss in children from high-income groups (Helvik et al., 2009; WHO, 2013). Adults from low-income nations were found to have a 48% prevalence of hearing loss, compared to high income groups which displayed a 34% prevalence of hearing loss (WHO, 2013). A clear correlation between hearing loss and age has also been demonstrated. The incidence of hearing loss at birth is only 0.1%, while approximate 1/3 of all individuals over 65 experience hearing loss (WHO, 2013; Olusanya et al., 2014). The high global prevalence of disabling hearing loss presents an area in clear need for the development of new effective treatments (WHO, 2013).

**Figure 1:**
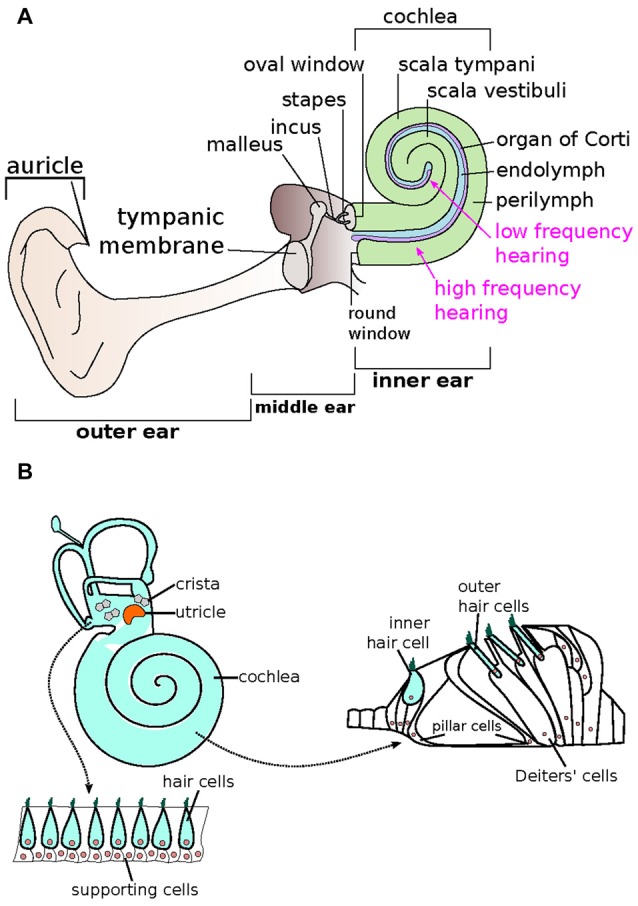
Schematic representation of the auditory system. **(A)** The human ear is composed of three sections—the outer, middle and inner ears. The inner ear is made up of the spiral-shaped cochlea, where endolymph (blue) and perilymph (green) reside. The organ of Corti (purple), responsible for relaying sound via specialized hair cells, is arranged tonotopically, where high (base) and low (apex) frequencies are processed in separate locations. (Adapted from Géléoc and Holt, 2014). **(B)** The mammalian inner ear consists of two types of sensory receptor organs, which include the hair cells (light blue) and supporting cells (white). The hair cells that make up the auditory sensory epithelia in the cochlea is also known as the organ of Corti, whereas the saccule, utricle and cristae make up the vestibular epithelia. The vestibular epithelia include alternating outer hair cells and supporting cells. In the organ of Corti within the cochlea, one row of inner hair cells is associated with three rows of outer hair cells. Supporting cells such as the pillar and Deiters’ cells make up the rest of the organ of Corti. (Adapted from Bermingham-McDonogh and Reh, 2011).

The complexity of the auditory system is what makes hearing particularly vulnerable to damage (Moser and Starr, 2016; Mittal et al., 2017). Sound transduction begins at the pinna, which is responsible for the collection and funneling of sound-waves into the auditory canal (Yan and Liu, 2008). At the end of the external auditory canal, sound actuates the tympanic membrane. The tympanic membrane is connected to the bones of the middle ear and travels from malleus to incus to stapes (Figure 1A). The stapes are attached to a membrane on the cochlea known as the oval window. Within the cochlea, the endolymph filled *scala media* is the vital element of hearing (Figure 1A). Within the *scala media*, the organ of Corti converts the mechanical energy of sound waves into electrical signals that are transduced to the auditory pathway in the brain (Kazmierczak and Muller, 2012). It does so through the use of hair cells, which contain stereocilia that move in response to specific soundwave initiated fluid waves (Goutman et al., 2015; Figure 1B). Tip links on the stereocilia open ion channels as a result of their movement, allowing for the production of an action potential from the potassium-rich endolymph entering the stereocilia (Karavitaki and Corey, 2010; Verpy et al., 2011). In addition to the essential elements of the conduction pathway, the tensor tympani and the stapedius muscle are responsible for damping the movement of the middle ear bones in response to high levels of sound. The contraction of these muscles is vital for preventing mechanical damage and stimulus-induced hearing loss. Failure in any of the elements of the hearing pathway, including the tensor tympani and stapedius muscles, may result in hearing loss (Yan and Liu, 2008; Géléoc and Holt, 2014; Moser and Starr, 2016; Mittal et al., 2017).

Hearing loss arises from a diverse array of etiologies, including over stimulation of sensory hair cells, trauma to the head, nerve impingement, ototoxic drugs, autoimmune disease and gene mutations (Mijovic et al., 2013; Furness, 2015; Goodall and Siddiq, 2015; Mittal et al., 2015, 2016; Venkatesh et al., 2015; Ben Said et al., 2016; van As et al., 2016). The goal of this review article is to summarize the recent advancements in restoring the hearing function employing new technologies (Table 1). We will also discuss the progress of genome editing technologies, including CRISPR/Cas9, as it might be applied to restore hearing by correcting the deleterious effect of gene mutations and for the restoration of normal physiological function.

**Table 1:** Summary of treatments and techniques for the restoration of hearing loss.

Restoration classification	Restoration technique	Summary of technique	Technique application	Advantages	Disadvantages	References
Gene therapy	Application of Hyaluronic acid to round window membrane	Applying hyalurinoic acid to the round window membrane, increased the permeability of the membrane to virus vectors.	Any pathology treatable by viral vector gene therapy.	This finding makes delivery of virus vectors more feasible.	Requires further research into gene therapy before this can be useful.	Shibata et al. (2012)
Gene therapy	*GJB2* gene delivery	Gjb2 was delivered through the use of an adeno-virus vector to mice with disorders in the gene.	Suitable for congenital hearing loss due to *gjb-2* deficiency	Prevented hearing loss in mice with GJB2 gene mutations. Presents the possibility of treatment of other genetic issues	Very specific to one gene. Gene must be delivered early in development.	Iizuka et al. (2015)
Gene therapy	Atoh-1 delivery	Delivery of Atoh-1 through adenovector has been found to induce recovery of hair cells.	Mouse models with aminoglycoside-induced ototoxicity damage.	Mouse models displayed a high level of recovery following damage. This modality could serve as a treatment for ototoxicity in mature organisms.	Thus far, studies have been limited to mouse models with aminoglycoside-induced ototoxicity	Baker et al. (2009)
Gene therapy	*MsrB3* gene delivery	Delivery of *MsrB3* gene into the oocyst of a mouse lacking the gene was found to result in normal stereocilliary bundles	Mice with congenital defects of Msr protein	Results in normal development of stereocilliary bundles	Tested only in mice. Specific for only *MsrB3* negative mice.	Kim et al. (2016)
Gene therapy	VGLUT3 delivery	Vesicular glutamine transporter 3 (VGULT3) deficiency is a cause of congenital deafness. Adenoviral delivery of the gene prevents the disease in mice	Mice with congenital deafness due to VLUT3 deficiency	Provides complete recovery in mice with the disease after 2 weeks of treatment	Tested only in mice. Specific for only VGLUT3 negative mice	Akil et al. (2012)
Gene therapy	GDNF overexpression	Glial cell line-derived neurotrophic factor overexpression can protect hair cells from ototoxicity due to gentamicin	Protective for individuals taking gentamicin	Removes a dangerous side effect of gentamicin	An extreme strategy to avoid one side effect of gentamicin. Has only been tested in mice.	Suzuki et al. (2000)
Stem cell	Stem cell therapy	Currently, stem cell therapy is in the early stages. If researchers are able to find a feasible method of stem cell differentiation and delivery, stem cells could serve as a promising new treatment.	Pathologies that have caused damage to the hair cells, most notably age and trauma induced hearing loss.	Generation of new stem cells that are more receptive and finely tuned than machine alternatives.	Current stem cell techniques are a long way from practical application. Yields of hair cells from stem cells are too low, and there is no viable delivery technique as of yet.	Géléoc and Holt (2014)
Molecular therapies	Antisense oligonucleotide	Antisense oligonucleotides were administered to mice in the early stages of development.	Usher syndrome 1c when administered early.	*In vivo* prevention of Usher syndrome 1c.	Treatment must be administered early in development. Treatment has not been tested on humans.	Lentz et al. (2013)
Molecular therapies	Clarin-1 gene stabilizers	Small molecules capable of stabilizing the clarin-1 gene.	Usher syndrome III in mice.	Clarin-1 gene stabilizers were found to prevent progressive hearing loss in CLRN1 USH3 mice.	Treatment has not yet been tested in humans.	Alagramam et al. (2016)
Molecular therapies	Wnt pathway activation	Wnt pathway has been found to stimulate stem cell differentiation, and thus the production of hair cells and progenitor cells.	Pathologies that have caused damage to the hair cells, most notably age and trauma induced hearing loss.	Induction of hair cell regeneration could lead to restoration of hearing loss.	There have been no *in vivo* experiments thus far.	Bramhall et al. (2014) and Cox et al. (2014)
Molecular therapies	γ-secretase inhibition.	γ-secretase was found to inhibit the differentiation of progenitor cells into hair cells. Inhibition of γ-secretase was found to increase progenitor progression into hair cells.	Pathologies in which hair cells fail to develop from progenitor cells. For the most part, congenital hearing disorders.	Full recovery of functional hair cells in mouse models.	No testing in humans thus far. Administration of inhibitors must be done early in development and must be applied directly to the cochlea.	Jeon et al. (2011)
Molecular therapies	Retinoblastoma inhibitors	Inhibition of retinoblastoma was found to cause progression of mature hair cells into mitosis.	Pathologies that have caused damage to the hair cells, most notably age and trauma induced hearing loss.	Increase in number of functional hair cells.	Patient must have viable, mature hair cells. Increased risk for tumors and apoptosis.	Sage et al. (2005)
Genome editing strategies	Gene-editing modalities.	Zinc finger nucleases, transcriptional activator-like effector nucleases, and CRISPR/Cas9 may be used to edit the genes that are malfunctioned in congenital hearing loss.	Congenital hearing loss.	Direct, point control of congenital hearing loss.	Currently no viable strategy for implementing genome editing for hearing loss.	Zou et al. (2015)

## Specialized Sensory Cell Regeneration

Many modern approaches to hearing restoration focus on the regeneration of the specialized inner ear sensory neuroepithelial cells found on the basilar membrane of the organ of Corti (Basch et al., 2016). The organ of Corti is a complex epithelial structure that utilizes hair cells to produce the action potentials that relay encoded sound to the auditory nerve and then to the central auditory pathway (Goutman et al., 2015). Development of this sensory epithelium involves the differentiation of cells from the cochlear epithelium into stromal cells and parenchymal sensory cells (Barr-Gillespie, 2015; Basch et al., 2016). The supporting cells are vital for the regulation of the specialized sensory cells, and there is evidence that some of these supporting cells may become specialized sensory cells when exposed to the right sequence of chemical signaling (Géléoc and Holt, 2014; Sienknecht, 2015; Whitfield, 2015). Notch and Wnt signaling are the two predominant pathways that play a crucial role in hair cell differentiation (Mizutari et al., 2013; Li et al., 2015). These two pathways determine the fate which cells will differentiate into hair cells, supporting cells or neurons in the cochlea (Żak et al., 2015; Waqas et al., 2016). Therefore, these two pathways have been targeted to develop hair cell regeneration therapies. Studies of hair cell regeneration center on three approaches: (1) generation of hair cells from intrinsic stem cells; (2) generation of hair cells from existing cochlear supporting cells; and (3) induction of cellular replication followed by maturation of both hair cells and support cells (Géléoc and Holt, 2014).

Stem cell differentiation is currently an immensely popular field of study for hearing restoration, but only moderate results have been achieved with this approach (Hu and Ulfendahl, 2013; Géléoc and Holt, 2014). Two issues arise from a stem cell-based regeneration approach. The first is that experiments thus far have produced hair cell like cells, but it is unclear if they have actually produced hair cells. Hair cell like cells differ from normal hair cells as they are generated using stem cells, *in vitro*. Although these hair cell like cells express markers expressed by normal hair cells, the functionality of these hair cell like cells has yet to be determined. One exception to this has been leucine-rich repeat-containing G-protein coupled receptor 5 (LGR5)-positive cells, which have been found to grow into fully functional hair cells. The finding that LGR5 positive cells were capable of differentiating into hair cells was an important one, but initial studies found that the differentiated cells were low in number, and died quickly after their initial differentiation and maturation (Shi et al., 2012; Bramhall et al., 2014; Cox et al., 2014). This initial finding led to a number of studies that sought to create hair cells with greater success. Control of the *Notch* and Wnt pathways were found to be effective for this goal. The inhibition of *Notch* signaling (and thus activation of the Wnt pathway) through the use of small molecule inhibitors resulted in differentiation of LGR5 positive cells into hair cells (Bramhall et al., 2014). Another study demonstrated that inhibition of *Notch* could induce differentiation of stem cells into hair cells even without the activation of the Wnt pathway (Li et al., 2015). β-catenin, another Wnt activator, was also found to generate hair cells *in vivo*, as was the hair cell fate determination factor Atoh1 (Kuo et al., 2015). Further investigations into the field of stem cell differentiation may yield more clinically applicable therapeutics. Mouse models with fluorescence ubiquitination cell cycle indicator (FUCCI) markers (Zielke and Edgar, 2015; Koh et al., 2017) and Lgr5-GFP positive cells (Bramhall et al., 2014), have been used to find small molecules capable of inducing stem cell differentiation into hair cell progenitors. Through the use of these models, a small molecule CHIR99021, a GSK inhibitor capable of inducing cochlear progenitor cells has been identified (Roccio et al., 2015). This finding presents the possibility of a valuable assay for harnessing the potential of stem cells for the design of treatment modalities for hearing loss.

A study attempted one of the most promising breakthroughs into the quantitative limitation of stem cell differentiation to create inner ear sensory epithelia through inducing step-wise differentiation in mouse embryonic stem cells (Koehler et al., 2013). By maintaining tight temporal regulation of bone morphogenetic protein (BMP), transforming growth factor-beta (TGFβ), and fibroblast growth factor (FGF), it was possible to generate otic placode-like epithelia that could differentiate into a sensory epithelium with cells possessing stereocilia bundles and kinocilia. The yield was approximately 1500 vestibular-like hair cells/aggregate. These differentiated cells displayed mechanoreceptive capacities and to be capable of synapsing with sensory neurons that were also created within the embryonic stem cell cultures. This development was accomplished by activating BMP while inactivating TGFβ to induce non-neural ectoderm, then inhibiting BMP and activating FGF to allow for induction of a pre-placodal epithelium (Koehler et al., 2013).

Development of hair cells from cochlear supporting cells is the most promising method of hair cell regeneration. An *in vitro* study demonstrated that cell cycle manipulation through the use of pharmacologic agents allows for the generation of hair cells from supporting cells. By generating P27^kip1^ (a cell cycle inhibitor) producing cells, it was possible to induce the production of a small number of hair cells from supporting cells (White et al., 2006). More exciting is the discovery that supporting cells can readily be transformed into hair cells with the use of γ-secretase (Mizutari et al., 2013). Although this study found shortcomings, such as the need to directly inject γ-secretase into the inner ear, the fact that this was an *in vivo* study that restored hearing in mice shows the promise of this approach for hearing restoration in humans.

The induction of mitosis in mature hair cells is currently the least regarded option. The only pathway of manipulation found thus far is the retinoblastoma pathway (Sage et al., 2005). Retinoblastoma protein was found to inhibit progression of mature hair cells into the cell cycle. Inhibition of retinoblastoma was found to induce the production of functional hair cells through mitosis (Mantela et al., 2005; Chen, 2006; Yu et al., 2010). A recent study described the generation of a dominant-negative (DN) retinoblastoma transgenic mouse model (Tarang et al., 2015). The characterization of this mouse model revealed downregulation of retinoblastoma in a number of systems including inner ear. Supernumerary inner HCs (IHCs) were demonstrable in P10 and P28 cochlea especially in the middle and basal turns of the cochlea. However, the manipulation of the retinoblastoma pathway presents the risk of tumors and apoptosis, and further studies are warranted to determine its applicability in hair cell regeneration.

Kremen1, a single pass transmembrane protein, is believed to influence hair cell fate specification (Nakamura et al., 2008). Kremen1 is a receptor protein for dickkopf (DKK), which act as antagonists to the WNT signaling pathway. DKK family proteins binds to Kremen 1 as part of the WNT inhibitory complex, resulting in the blockage of WNT signaling and the ultimate β-catenin degradation (Nakamura et al., 2008). A recent study evaluated different expression pattern effects of Kremen1 in the prosensory domain of a developing mouse cochlea as well as in the zebrafish lateral line. Using gain- and loss-of-function mutations, it was found that over expression of Kremen1 drastically inhibited the development of hair cells, and restricted the affected cells to develop into supporting cells. Similarly, decreased levels of Kremen1 resulted in affected cells to take on a hair cell fate, presumably through canonical Wnt signaling that is required for hair cell formation (Mulvaney et al., 2016). Further studies will help in further deciphering the role of Kremen1 in hair cell regeneration and developing novel treatment modalities.

## Molecular Approaches to Treatment of Hearing Loss and Usher Syndrome

While there are currently no molecular treatments available for hearing loss, molecular approaches have revealed a wealth of information that pertains to hearing loss. The most promising findings have been centered on the treatment of the rare genetic disease, Usher’s syndrome. Usher syndrome is the world’s most prominent cause of deaf-blindness (Friedman et al., 2011; Bonnet and El-Amraoui, 2012; El-Amraoui and Petit, 2014; Mathur and Yang, 2015; Hartel et al., 2017). The blindness that results from the disease is a form of retinitis pigmentosa, while the deafness is variable depending on the involved genes (Sun et al., 2016). There are three classifications of Usher syndrome, each one resulting from mutations affecting a unique set of genes (Yan and Liu, 2010). Usher syndrome I deafness starts from birth and is the result of defects in genes that regulate the development of stereocilia. These genes include *CDH23*, *USH1C*, *USH1G*, *MYO7A* and *PCDH15* (Bork et al., 2001; Ahmed et al., 2003; Yan and Liu, 2010; Ben-Rebeh et al., 2016). In Usher syndrome II patients, there is difficulty in hearing rather than deafness, and is the result of mutations in *DFNB31*, *GPR98* and *USH2A* (Ahmed et al., 2003; Yan and Liu, 2010). Usher syndrome III is characterized by progressive hearing loss associated with mutations in *CLRN1* that encodes clarin-1 protein (Ahmed et al., 2003; Yan and Liu, 2010; Gopal et al., 2015; Alagramam et al., 2016).

A recent study investigated the potential of treating a mouse model of Usher syndrome with antisense oligonucleotides (ASO; Lentz et al., 2013). In these models, harmonin was found to be truncated. By using specially-designed ASO to replace the mutated c.216G>A nucleic acid in the USH1C gene via the correction of damaged pre-MRNA splicing of transcripts, it was possible to prevent hearing loss in these mice (Lentz et al., 2013). Administration of one ASO dosage minimized Usch1c c.216G>A splicing, improved the organizational structure of cochlear sensory epithelial cells, and improved damaged hair and vestibular cells while improving low-frequency hearing in *in vivo* mouse models (Lentz et al., 2013). This finding was seen to be useful in the prevention of Usher syndrome 1c when administered in the early stages of development, but not the reversal of the syndrome (Lentz et al., 2013). In another study, the development of Usher syndrome III in mice was prevented by stabilizing the gene for clarin-1 through the use of small molecule, BF844 (Alagramam et al., 2016). The only mutation site known in Usher syndrome III is the gene coding for clarin-1. High throughput screening has been utilized to identify small molecules that could be used to stabilize the *CLRN1^N48K^* gene. BF844 was found to stabilize the *CLRN1^N48K^* gene, and as such, was seen to prevent the degradation of hearing that is associated with Usher syndrome III. *CLRN1^N48K^* USH3 mouse models displayed an increase in hearing of three orders of magnitude when treated with the BF844 molecule, and this treatment was seen to prevent hearing loss when applied before the onset of loss and at the early stages of deafness (Alagramam et al., 2016).

Outside of the treatment of Usher syndrome, molecular studies have mostly been useful only in uncovering information about the generation of hair cells *in vitro*. Physiological conditions do not allow for the regeneration of hair cells, but it is hypothesized that pharmaceutical agents that target certain genes may result in a regeneration of hair cells. *In vitro* studies of cochlear supporting cells found that induction of the cell cycle through the use of pharmaceuticals may work to induce the regeneration of hair cells. The manipulation of the Wnt signaling pathway can result in the differentiation of endogenous stem cells into inner ear sensory epithelial cells (Bramhall et al., 2014; Cox et al., 2014). A similar effect has been observed with γ-secretase, where inhibition led to increased differentiation of progenitor cells into hair cells (Jeon et al., 2011). Understanding the host-signaling pathway that drives the differentiation of hair cells has the potential to provide novel avenues of treatment for hearing loss.

Deafness associated with the loss of sensory hair cells results in progressive pathophysiological changes leading to the degeneration of most auditory neurons (Moser and Starr, 2016). It is now possible to consider these events in the broader context of anti-apoptotic survival factors in the peripheral and central nervous systems. When a neuron is de-afferenated, a loss of neurotrophin proteins changes the oxidative state of biological molecules and leads to the creation of free radicals. The formation of free radicals then alters intracellular levels of Ca^2+^, increasing expression levels of pro-apoptotic genes (Miller et al., 2002). This degeneration can be prevented by applying chronic electrical stimulation (Miller et al., 2002). The administration of the potent neurotoxin tetrodotoxin *in vivo* can prevent the effects of electrical stimulation, which shows that Ca^2+^ mediated action potentials are an integral part of the rescue process. The effects of electrical stimulation can also be blocked by Verapamil, a calcium channel blocker, indicating the need of L-type calcium channel activation for stimulus-driven survival of auditory neurons (Miller et al., 2002). Administration of electrical stimulation can be delayed and can still be of benefit to auditory nerve neurons, even after the start of neuronal apoptosis. Other studies have also demonstrated that neurotrophic factor intervention restores auditory function as well as preserves nerve survival and electrophysiological responsiveness in experimental animal models of hearing loss (Shinohara et al., 2002; Yamagata et al., 2004; Sly et al., 2012; Leake et al., 2013). The “neurotrophic factor hypothesis” which is used to explain the effects of aging and neurodegeneration by contending developing neurons compete for limited neurotrophic factor supplies can thus be supported by these studies (Miller et al., 2002; Khalin et al., 2015). With the advancement of new technologies including gene therapy, it will be possible to efficiently deliver the neurotrophic factors into the inner ear to prevent hearing loss.

## Gene Therapy

Hearing loss can be caused by both environmental and genetic etiological factors, with the genetic contribution comprising for the majority of the cases (Yan and Liu, 2008; Mittal et al., 2015, 2016; Bolz, 2016; Xia W. et al., 2016). To develop therapies for hearing loss due to genetic etiology, methods for gene transfer into the inner ear by *in vivo* electroporation have been developed (Wang et al., 2012). In a study, E11.5 otocyst were transfected with a plasmid encoding enhanced green fluorescent protein (EGFP). Whole mount immunostaining of the cochlea from a postnatal day 6 (P6) pup whose otocyst was injected and electroporated at E11.5 demonstrated EGFP expression from the base through the middle turn of the cochlea showing efficient gene transfer.

**Figure 2:**
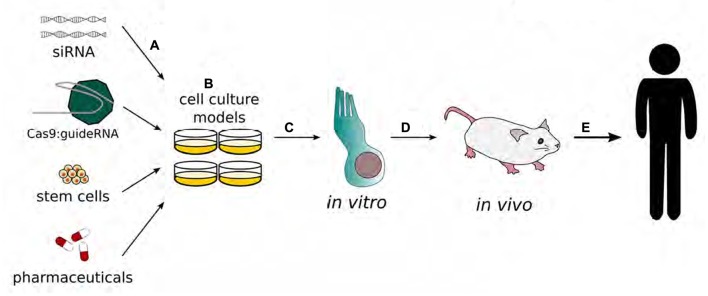
Schematic representation of the development of gene therapy in the inner ear. Gene therapy begins with transfection of adenovirus vectors **(A)** or drug applications **(B)** to cell cultures. Modified functions are evaluated in *in vitro* models, specifically inner and/or outer hair cells. **(C)** The results with the best outcomes will move on to be tested in *in vivo* systems **(D)** to validate therapeutic benefits before being tested in human clinical trials **(E)**. (Adapted from Rousset et al., 2015).

For many ethnic populations, the most prevalent form of genetic deafness is caused by recessive mutations in the gene for a gap junction protein, i.e., beta 2, 26 kDa (GJB2), which is also known as connexin 26 (Cx26; Banjara et al., 2016; Du et al., 2016; Mielczarek et al., 2016; Moctar et al., 2016; Xia H. et al., 2016). A gene delivery system has been used to rescue hearing in a mouse model of *GJB2* deletion (Iizuka et al., 2015). Following *GJB2* gene deletion, gene delivery systems have been used to replace mutations and improve hearing function in mouse models (Iizuka et al., 2015). A mutation of the Cx26 (*GJB2*) gene in mice results in the improper development of cochlear sensory epithelial cells and profound sensorineural deafness. An Adeno-associated viral (AAV) vector can be used to replace a mutated Cx26 with a normal copy of the gene in order to improve hearing function and cochlear development. The use of viral vectors and other methods to replace Cx26 genes and improve hearing function in mouse models could thus result in the creation of a new gene-based therapies to treat hearing loss due to genetic etiology (Iizuka et al., 2015; Figure 2).

In order to repair lost function to damaged inner ear neuroepithelium, several therapeutic strategies have been suggested (Gubbels et al., 2008; Brigande et al., 2009; Jiang et al., 2013; Depreux et al., 2016; Gomes et al., 2016). Hair cells damaged from vestibular aminoglycoside ototoxicity in mouse models were regenerated using an adeno-vector delivery system to promote genes that express Atoh1 (Baker et al., 2009). Additionally, mice that received the Atoh1 gene therapy showed an improvement in their vestibular function. A number of other studies have demonstrated that administration of Atoh1 gene therapy to the cochlea of embryonic animals (Woods et al., 2004; Gubbels et al., 2008), neonatal animals (Shou et al., 2003; Chen et al., 2013) and mature animals (Izumikawa et al., 2005; Yang et al., 2012; Atkinson et al., 2014) lead to hair cell regeneration though at varying degrees. Although very few treatment options exist for the management of vestibular disorders, hair cell regeneration may serve as a platform to improve these conditions. Development of Atoh1-based gene therapy for vestibular hair cell loss may provide an initial opportunity for developing gene therapy for inner ear diseases.

**Figure 3:**
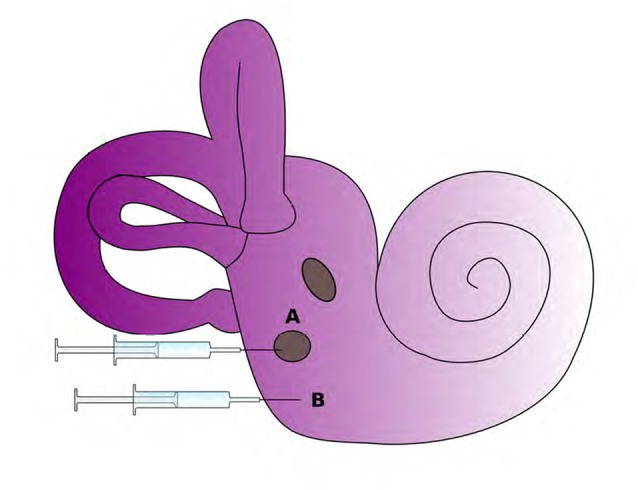
Potential routes for delivery of therapeutics to the inner ear. The round window membrane **(A)** is accessible from the middle ear space or delivery via cochleostomy **(B)** is also possible as it directly accesses to inner ear tissues. (Adapted from Rousset et al., 2015).

Cochlear gene therapy might provide a new avenue for the treatment of severe hearing loss by inducing regeneration or phenotypic rescue. One necessary step to establish this therapy is the development of a safe and feasible method and site of delivery of this type of therapy, ideally without requiring drilling the bony wall of the cochlea (Figure 3). Viral vectors are generally impermeable to the round window membrane (RWM) and are unable to cross into the scala tympani perilymph and cochlear epithelial cells. Hyaluronic acid (HA), a nontoxic and biodegradable reagent, has been used to increase RWM permeability to viral vectors in guinea pig models. HA is applied to RWMs in guinea pig models in order to increase inner ear permeability before adeno-viruses deliver an eGFP reporter gene (Ad-eGFP) to cochlear tissues (Shibata et al., 2012). The introduction of transgenes to repair damaged cochlear hair cells is made more feasible by increasing the permeability of the RWM to viral vectors through the usage of HA. The usage of HA is therefore an atraumatic therapy that can potentially improve research methods and future clinical applications (Shibata et al., 2012).

Another important protein associated with hearing in mammals is methionine sulfoxide reductase B3 (MsrB3). MsrB3 stereo-specifically repairs methionine-R-sulfoxide, which is an altered protein created by reactive oxygen species that can lead to inner ear tissue damage (Ahmed et al., 2011; Kwon et al., 2014). A down-regulation of *MsrB3* gene was found to cause severe to profound congenital hearing loss due to the damage of inner ear sensory epithelia. Down-regulation of *MsrB3* leads to a disorganization of inner ear cells, leading to epithelial cell damage and ultimate cellular apoptosis (Shen et al., 2015). In mice models, *MsrB3* gene can be delivered to developing embryos using transgene therapy. Through the use of a trans-uterine delivery method, an adenovirus vector can deliver *MsrB3* gene around embryonic day 12.5 into *MsrB3* (−/−) mouse otocyst. Treated mice have demonstrated hearing recovery on postnatal day 28, and *MsrB3* mRNA as well as protein expression was demonstrable in the cochlear extracts. Additionally, the morphology of the stereociliary bundles in the rescued ears of *MsrB3*(−/−) mice were similar to those in normal *MsrB3*(+/+) animals (Kim et al., 2016).

Glutamate receptors are involved in the hearing function (Seal and Edwards, 2006; Obholzer et al., 2008; Lee et al., 2015; Münster-Wandowski et al., 2016). Congenital deafness is also observed in mice models that lack a vesicular glutamate transporter-3 (VGLUT3). This hearing loss is a result of a decrease in glutamate release at the inner hair cell afferent synapse (Ruel et al., 2008; Seal et al., 2008). When using an adeno-associated virus type 1 (AAV1) as a transgenic vector to deliver VGLUT3 to the cochlea, expression of VGLUT3 is only seen in inner hair cells rather than in all sensory epithelia. (Akil et al., 2012). There was a partial repair of the unconscious startle reflex and a normalization of the auditory brainstem response threshold that was observed 2 weeks after AAV1-VGLUT3 delivery. After the transgenic delivery of VGLUT3, the IHC ribbon synapses of mutant mice also showed improvement in neurotransmission for auditory function. The results of this study demonstrate a successful restoration of hearing in mice by the utilization of transgenic techniques, which represents a significant advance towards the development and application of gene therapy for the treatment of human deafness.

Inner ear sensory epithelial cells are often damaged or lost after the use of the antibiotic gentamicin (Suzuki et al., 2000; Jiang et al., 2016). Adenovirus-mediated overexpression of glial cell line-derived neurotrophic factor (GDNF) can be used to counter this ototoxicity and inner ear injury (Suzuki et al., 2000). A study determined the protective effects of adenovirus-mediated overexpression of GDNF against gentamicin ototoxicity (Suzuki et al., 2000). The results of this study have demonstrated that this therapy can rescue the inner ear structure and function against damage caused by exposure to an ototoxic level of gentamicin (Suzuki et al., 2000).

Recently, there has been an increased interest in exploring the potential of brain derived neurotrophic factor (BDNF) to rescue hearing using adenovirus. In one study, guinea pigs were exposed to noise to induce cochlear damage (Zhai et al., 2011). Guinea pig cochleas were infused with recombinant adenovirus vector expressing BDNF or LacZ recombinant adenovirus vector or artificial perilymph. A significant decrease in ABR thresholds were observed in animals that received BDNF vector compared to LacZ or artificial perilymph group at 8-weeks of cochlear perfusion. In BDNF vector treated group, there was a strong expression of BDNF in all turns of cochlea including the spiral ligament, labium limbi tympanicum and supporting cells. The number of spiral ganglion in BDNF treated group were significantly higher than in LacZ or artificial perilymph treated animals. The results of this study suggest that BDNF exerts protective effects on spiral ganglions in response to cochlear insults such as noise leading to rescue of hearing. However, due to immunogenic and inflammatory potential of adenoviruses, there is a need to design better viral vectors for gene therapy.

AAV vectors are currently the most preferred choice for delivering inner ear gene therapy, due to their limited toxic and immunogenic effects (Shu et al., 2016; Landegger et al., 2017). Different serotypes of these vectors have been explored, e.g., in an *in vivo* experiment, AAV3 was found to transduce cochlear IHCs in the basal and middle cochlear regions exclusively with notable efficiency (Lalwani et al., 1996). Another study looked at five AAV vectors of serotypes 1, 2, 5, 6 and 8, and upon injection through the scala media in both normal and severely damaged inner ears, sensory transduction was observed within most cells that make up the inner ear, including sensory hair cells, supporting cells, the auditory nerve, and spiral ligament cells (Kilpatrick et al., 2011). Most recently, 12 different AAV vectors of different serotypes were injected into neonatal and adult mice *in vivo*, each resulting in cell type specificities and expression levels, as measured by expression of a reporter gene (GFP; Shu et al., 2016). Ten of these serotypes were found to infect neonatal inner ears and eight infected adult inner ears. The hair cells and supporting cells had higher affinities for AAV infection, which makes this method ideal. Unfortunately, it was also noted that while neonatal inner ear delivery of AAV vectors did not adversely affect hearing, adult inner ear injections ultimately resulted in outer hair cell death and a subsequent hearing loss (Shu et al., 2016). Furthermore, the use of AAVs is restricted by the size limit available for inserts (i.e., less than 4.7 kb; Akil et al., 2012). Recently, it has been demonstrated that exosome-associated AAV vectors (exo-AAVs) are efficient carriers of transgenes into cochlear and vestibular hair cells both *in vitro* and *in vivo* (György et al., 2017). Exo-AAV vectors outperform conventional AAV vectors in gene transfer efficiency and are also well tolerated by inner ear tissues. Another study developed a synthetic AAV vector, i.e., Anc80L65, and demonstrated that round window membrane injection of this synthetic vector into mice enables safe and efficient gene transfer to the mammalian inner ear (Landegger et al., 2017). Highly efficient transduction of both inner and outer hair cells was observed in mice. Round window membrane injection was also well tolerated, as indicated by hearing and vestibular function and immunologic parameters. Another study demonstrated that early reintroduction of wild-type harmonin using this synthetic AAV vector, Anc80L65, can lead to recovery of gene and protein expression, restoration of sensory cell function, rescue of complex auditory function and recovery of hearing and balance behavior to near wild-type levels in a mouse model of Usher syndrome type 1c (Pan et al., 2017). There is a hope that AAV will develop to be a central vector to deliver gene therapy for hearing deficits, but there are still significant improvements needed before gene delivery can be a safe and efficient way to deliver therapy to the inner ear.

## Stem Cell Therapies

Besides gene therapy, stem cell therapy is an emerging technology with potential applications for the treatment of human diseases. The use of stem cells to assist in the repair of damaged cardiac tissue following a heart attack, to accelerate wound healing in diabetic patients, and treat diseases of the eyes are just a few of the clinical applications of the rapidly developing field of stem cell therapy. The inner ear can be made accessible to placement of exogenous stem cell in the scala tympani or scala media of the cochlea, Rosenthal’s canal where the spiral ganglion resides, and into the areas of the vestibular sensory receptors via direct injection through a micro-syringe. Stem cells have the potential to contribute to the repair and restoration of inner ear sensory structures and their sensory ganglia. Stem cells provide protection via direct integration into the damaged receptor or ganglion with the subsequent differentiation of the transplanted stem cells into replacement sensory hair or neuronal cell types.

Using sphere culture methods, a series of experiments demonstrated that both neonatal cochlear and vestibular tissues contained endogenous stem cells. These cells possess the potential to generate hair cell like cells, but only vestibular tissue obtained from adult animals retained the potential to form hair cell like cells within cultured spheres (Oshima et al., 2007, 2009). An *in vitro* study has shown that murine embryonic stem cells (ESCs) can be induced to initiate stepwise differentiation of both inner ear hair cells and neurons (Koehler et al., 2013). The hair cells formed from these ESCs in a 3-D culture system exhibit functional characteristics of hair cells. In addition, these cells formed specialized synaptic contacts with the sensory neurons that developed within these cultures (Koehler et al., 2013). Further studies of ESCs in the 3-D culture system has revealed that modulation of Wnt signaling with a potent Wnt agonist can enhance the process of differentiation of inner ear sensory tissues within these cultures (DeJonge et al., 2016). These *in vitro* findings with ESC cultures (Koehler et al., 2013; DeJonge et al., 2016) are in agreement with results from a series of experiments which examined the role that a gene target of Wnt signaling (Lgr5) plays in determining the stemness of support cells located within the neonatal cochlea (Chai et al., 2012; Shi et al., 2012).

A lineage tracing study in neonatal mice has shown that Lgr5 positive inner pillar cells and 3rd row Deiter’s cells are the cell types that predominantly produce new outer hair cells (Bramhall et al., 2014). Cells positive for Lgr5 expression were also isolated from neonatal vestibular tissues and were associated with determining the stemness of support cells and their ability to produce new hair cells but these Lgr5 positive cells did not produce any neuronal cells (McLean et al., 2016). This same study revealed that PLP-1 positive glial cell derived from neonatal spiral ganglia explants could act as a neuronal progenitor source and give rise to sensory neurons, glia and oligodendrocytes but not to any hair cells (McLean et al., 2016).

An *in vitro* study of neural stem cells (NSCs) found within the spiral ganglion identified micro-RNA 124 as a key factor in promoting both neuronal differentiation and neurite outgrowth from the spiral ganglion’s NSCs (Jiang et al., 2016). Results from a rat model of spiral ganglion neuronal degeneration also point to the importance of damage-initiated cochlear Schwann cell secretion of Wnt-1 in enhancing the survival of transplanted NSCs and their differentiation into replacement neurons within the sensory ganglion (He et al., 2014). Another type of stem cell considered as a possible candidate to replace lost spiral ganglion neurons are bone marrow derived mesenchymal stem cells (MSCs; Matsuoka et al., 2007). Experiments in gerbils showed that the direct injection of MSCs into the modiolus of an injured cochlea resulted in the successful transplantation of these stem cells but that injection of MSCs into the scala tympani did not result in the transplantation of the injected cell into the modiolus (Matsuoka et al., 2007).

Induced pluripotent stem cells (iPSCs) of both mouse and human origins have also been evaluated as potential replacement therapies for lost spiral ganglion neurons and both of these iPSC cell types have been shown to express neuronal phenotypes and vesicular glutamate transporter one (VGLUT-1) which identifies glutamatergic neurons (Nishimura et al., 2009; Ishikawa et al., 2015). In addition to these findings, human iPSCs transformed into glutamatergic neurons transplanted into a guinea pig cochlea were found to survive this transplantation if the animal’s immune system was suppressed (Ishikawa et al., 2015). Human iPSCs can also be transformed into hair cell like cells *in vitro* through the use of a step-wise protocol and with the use of basic FGF (bFGF) as an inducing factor (Ohnishi et al., 2015). Of note, the yield of hair cell like cells from this protocol was very low and therefore is not as promising for hair cell replacement as it is for the replacement of spiral ganglion neurons.

NSCs obtained from olfactory epithelium (oe) is an important consideration because they can be obtained from a patient and then possibly used to treat that same patient. An animal study has determined that these oe-NSCs can be used to replace injured or lost spiral ganglion neurons in a rat model of hearing loss (Xu et al., 2016). An excellent source for human MSCs to be use in possible stem cell therapy to treat damaged inner ear sensory receptors and ganglia are cells derived from human umbilical cord: namely, Wharton’s Jelly cells, also known as human umbilical cord mesenchymal stromal cells (hUCMSCs; Mellott et al., 2016). One of the main advantages of hUCMSCs is that they retain their ability to differentiate into multiple cell types arising from all three embryonic germ layers (Mellott et al., 2016). These hUCMSCs when treated with a vector expressing Atoh 1 can be induced to differentiate into hair cell like cells that express hair-cell specific markers (Devarajan et al., 2013) making them a possible candidate for the treatment of both hearing loss and balance disorders.

One final approach to cover in the application of stem cells to the treatment of deafness is to use cell cultures of a patient’s own stem cells (iPSCs) to model their genetic disease. This approach helps in deciphering the underlying pathogenic mechanisms and then to test cutting edge gene editing methods including CRISPR/Cas9 to develop treatment modalities for the discovered genetic defect (Chen S. et al., 2016; Tang et al., 2016; Hosoya et al., 2017).

## Gene Editing Strategies

The genome editing technologies using programmable nucleases can modify the genome at a targeted locus and is recognized to be a valuable tool in studying the function of genes associated with hearing loss (Zou et al., 2015; Gurumurthy et al., 2016). These approaches have only recently been applied to study defects in the inner ear, but the goal is that genome editing 1 day could be optimized to restore wild-type sequences in native DNA thereby correcting for gene mutations that adversely affect hearing and balance. There are three major programmable nucleases, which include Zinc finger nucleases (ZFNs), transcriptional activator-like effector nucleases (TALENs), and CRISPR/Cas9 (Zou et al., 2015; Eid and Mahfouz, 2016; Govindan and Ramalingam, 2016; Mei et al., 2016; Torres-Ruiz and Rodriguez-Perales, 2016; Wang et al., 2016). These nucleases induce a site-specific DNA double-strand break. This break can then be repaired by either homologous or non-homologous mechanisms (Zou et al., 2015; Chandrasegaran and Carroll, 2016; Kim, 2016). Depending on the pathway of repair, one can introduce different mutations at a specific site. ZFNs or TALENs are able to be packaged into viral vectors, targeting supporting cells or hair cells (Gaj et al., 2013). However, the elegant and simple design of the CRISPR/Cas9 system makes this approach more favorable than the other two techniques (Zou et al., 2015; Gurumurthy et al., 2016). The functions of inner ear genes can be efficiently examined by disrupting normal gene alleles via non-homologous end-joining (NHEJ) mechanism in the CRISPR/Cas9 system (Zou et al., 2015). In terms of its validity as a treatment option, CRISPR/Cas9 has the potential to repair gene mutations via homology-directed repair or disrupting dominant mutations via NHEJ (Zou et al., 2015).

Traditional models to study hearing loss in transgenic animals can be laborious and expensive. CRISPR/Cas9 can create deletion models by producing embryonic stem cells exposed to NHEJ or single nucleotide mutation and gene insertion models via homology directed repair (An et al., 2016; Chen J. R. et al., 2016; Markossian and Flamant, 2016; Nakagawa et al., 2016; Wettstein et al., 2016). We have shown earlier that complexes formed from Cas9 and nucleic acid gRNA can be delivered to mouse inner hair cells directly (Zuris et al., 2015). In addition, these complexes were individually examined and were found to induce effective genome edits either by GFP signal knockdown in Atoh1-GFP transgenic mice or by detection of small insertions and deletions of short sequences (indels) via high-throughput sequencing (Zuris et al., 2015). Other advantages with this system are the possibility of editing multiple genes simultaneously as well as the minimal toxic effects when delivering the complex or during the actual editing of the genome. Cas9 and gRNAs have short half-lives, but the genome editing is permanent. Unfortunately, this method is at present limited to only outer hair cells and has not yet been optimized to target genes in inner hair cells effectively. However, there are still considerable concerns about potential off-target effects of editing the genome. There are still no acceptable methods for identifying or preventing the precise off targets effects of genome editing. Current strides are being made in implementing this pioneering technique and given the rapid pace in this field, these complications are likely to be overcome in the near future.

A recent study utilized the CRISPR/Cas9 knock-in method to confirm a mutation effect that was found in a classical genetic analysis (Miyasaka et al., 2016). The study used Jackson shaker (*Ush1g^js^*) as the mouse model for recessive deafness due to a homozygous mutation of *Ush1^g/Sans^* and found that heterozygous mice showed early-onset PHL (ePHL) along with progressive stereocilia degeneration involving the outer hair cells. Using traditional genetic analytical methods, they found that ePHL *in Ush1^gjs/+^* mice was linked with an interval in chromosome 10. This chromosome includes the gene for cadherin 23, another gene known to cause deafness in humans (Bork et al., 2001). Using the CRISPR/Cas9 knock-in system, mice homozygous for Ush1gjs and mice double heterozygous for an A to G (one base) substitution within the cadherin 23 gene. Ultimately, using this technology, their analyses revealed a relationship between the *Ush1g* and *Cdh23* genes, such that at least two mutant alleles within these genes will eventually manifest into ePHL (Miyasaka et al., 2016). Recently it has been demonstrated that CRISPR/Cas9-mediated HDR can successfully correct the *Cdh23^ahl^* allele in C57BL/6NTac mice, and rescue the associated auditory phenotype (Mianné et al., 2016). The heterozygous *Cdh23* (*Cdh23^ahl/753A >G^*) mice treated with CRISPR/Cas9 displayed normal hearing thresholds and a full complement of cochlear sensory hair cell stereocilia bundles compared to homozygous C57BL/6NTac mice (*Cdh23^ahl/ahl^*) at 36 weeks of age. It was demonstrated that the CRISPR/Cas9 approach is highly specific, with no lesions identified at any of the predicted off-target sites. These studies provide proof of principle that CRISPR/Cas9 genome editing can be utilized to correct mutations in deafness genes and has the ability to rescue hearing function.

The development of *in vitro* systems to substantiate gene therapies that were successful in animal models holds great potential for translational of these therapies from the research lab to the clinic. There has been some success in culturing human inner ear tissue in organotypic cultures that can be used to study potential treatment modalities (Kesser et al., 2007). Patients undergoing resection of tumors growing on the vestibulocochlear nerve also have viable vestibular tissue excised. Adenoviral vectors containing genes for wild-type GFP and potassium channel protein KCNQ4 were applied to the sensory epithelia. KCNQ4 is known to cause dominant-progressive hearing loss when mutated (Kubisch et al., 1999; Mittal et al., 2017). Strong expression of an exogenous transgene (GFP) in hair cells and supporting cells was observed, indicating that the adenovirus transfection can drive the expression of a wild-type form of human deafness genes, e.g., KCNQ4. This study ultimately presented an effective experimental way to evaluate gene therapies designed to restore hearing loss and is a useful transition to validate these therapies before human clinical trials can be designed and implemented.

## Conclusions

Recent advancements in technologies have opened up several promising avenues to combat hearing loss with a great translational potential. However, many challenges still exist that make it extremely difficult to properly regenerate hair cells. Proper orientation, tonotopic arrangement, appropriate integration, as well as adequate innervation of the cells all have to be considered to provide a functional solution to hearing loss. Of the different strategies outlined, gene will require considerable improvement in order to better control gene targeting, editing and expression. AAV are currently the popular choice in delivering these gene therapies, but with the advent of other more sophisticated gene editing technologies like the CRISPR/Cas9 system, it is likely that these newer strategies will also become extremely useful in sensory regeneration research. While the technical capability of these methods have been explored in supporting cells and inner hair cells, there has not been substantial understanding of pathologic mechanisms to advance these strategies into effective therapies. At most, inner ear gene therapy has largely focused on gene replacement or amplification, but these tactics are only effective in recessive deafness. Since some dominant forms of deafness result from single point mutations, gene-editing technologies including CRISPR/Cas9 will become a valuable tool to deliver therapy in the near future.

Due to limited ability of hair cells to regenerate, this aspect of hearing loss will continue to receive the most attention to develop novel treatment modalities. The main therapeutic strategies will tend to implicate resetting the course of the reprogramming of supporting cells to a proliferative progenitor state followed by a differentiation phase where both new hair cells and supporting cells are produced. One other stem cell option may in the future be the *ex vivo* expansion of a patient’s own stem cells and then reintroduction into a damage site such as spiral ganglion. Our comprehension of the underlying mechanisms is not yet complete enough to progress into tangible, working treatments. However, the auditory community working in the field of regenerative medicine is positive that with the evolution of new technologies, treatments for inner ear sensory disorders are sure to emerge.

## Author Contributions

RM, DN, APP, LHD, JM, DY, AAE, TRVW and XZL drafted and wrote the manuscript.

## Conflict of Interest Statement

The authors declare that the research was conducted in the absence of any commercial or financial relationships that could be construed as a potential conflict of interest.
